# ADAM9 expression in pancreatic cancer is associated with tumour type and is a prognostic factor in ductal adenocarcinoma

**DOI:** 10.1038/sj.bjc.6601645

**Published:** 2004-03-02

**Authors:** R Grützmann, J Lüttges, B Sipos, O Ammerpohl, F Dobrowolski, I Alldinger, S Kersting, D Ockert, R Koch, H Kalthoff, H K Schackert, H D Saeger, G Klöppel, C Pilarsky

**Affiliations:** 1 Department of Visceral, Thoracic and Vascular Surgery, University Hospital Carl Gustav Carus, Technical University of Dresden, Fetscherstrasse 74, Dresden 01307, Germany; 2 Department of Pathology, University of Kiel, Germany; 3 Molecular Oncology Research Group, Clinic for General Surgery and Thoracic Surgery, University of Kiel, Germany; 4 Institute of Medical Informatics and Biometrics, Technical University of Dresden, Germany; 5 Department of Surgical Research, University Hospital Carl Gustav Carus, Technical University of Dresden, Germany

**Keywords:** pancreatic cancer, ADAM9, gene profiling, immunohistochemistry, prognosis, survival

## Abstract

Gene expression profiling revealed ADAM9 to be distinctly overexpressed in pancreatic ductal adenocarcinoma (PDAC). We examined the relevance of ADAM9 expression in PDAC diagnosis and prognosis. A total of 59 infiltrating PDACs, 32 specimens from patients with chronic pancreatitis, 11 endocrine tumours and 24 acinar cell carcinomas were immunohistochemically analysed for ADAM9 expression. Staining for ADAM9 was detected in 58 out of 59 (98.3%) PDACs and in two out of 24 (8.3%) acinar cell carcinomas, but not in endocrine tumours. In the non-neoplastic pancreas, whether normal or chronically inflamed, ADAM9 was expressed in centroacinar and intralobular duct cells, but not in interlobular duct cells and their hyperplastic lesions. Pancreatic ductal adenocarcinomas showing cytoplasmic ADAM9 expression correlated with poor tumour differentiation and also with shorter overall survival than in cases showing only an apical membranous staining pattern (*P*=0.001). Multivariate analysis identified cytoplasmic ADAM9 expression as an independent marker of shortened survival in a set of 42 curatively (R0) resected PDAC (*P*<0.05, hazard ratio 2.85, 95% confidence interval: 1.21–6.71). The results show that ADAM9 expression distinguishes PDACs from other solid pancreatic tumours. In addition, cytoplasmic ADAM9 overexpression is associated with poor differentiation and shortened survival. Therefore, ADAM9 overexpression might contribute to the aggressiveness of PDACs.

Pancreatic ductal adenocarcinoma (PDAC) is an important cause of malignancy-related death. In the US it ranks fifth among the leading causes of cancer death, accounting for approximately 30 000 deaths annually (Jemal *et al*, 2003). Apart from surgery, there is no effective therapy and even resected patients frequently die within 1 year of the operation. In the past years, several genes have been identified as related to the development of PDAC (Hruban *et al*, 1998; Slebos *et al*, 2000). However, considering the complexity of the genome, it is most likely that most of the molecular changes causing PDAC still need to be elucidated (Bardeesy and DePinho, 2002). Moreover, there is still a need for prognostic markers in this devastating cancer disease.

Recently, others and we identified ADAM9 as one of the genes that is overexpressed in PDAC, when compared to normal pancreatic tissue using DNA microarray transcript profiling. This result was validated by an RT–PCR analysis in PDAC cell lines, which revealed ADAM9 expression in 13 of the 20 cell lines, and by immunohistochemistry in a small set of PDACs (Grutzmann *et al*, 2003; Iacobuzio-Donahue *et al*, 2003). ADAM9 overexpression was also demonstrated in prostate, breast and liver cell carcinomas (McCulloch *et al*, 2000; Le Pabic *et al*, 2003; O’Shea *et al*, 2003). ADAM9 is a member of the large ADAM family of proteases, which are type I transmembrane proteins with both metalloproteinase and disintegrin-containing extracellular domains. The ADAMs are implicated in the proteolytic processing of membrane-bound TNF*α* precursors and are involved in modulating cell–cell and cell–matrix interactions (Amour *et al*, 2002).

Although at present the precise molecular and biological mechanisms of ADAM9 remain to be elucidated, ADAM9 may be involved in the carcinogenesis of PDAC. For this reason, we were interested in determining whether the proposed differential expression of ADAM9 in PDAC at the RNA level could be confirmed at the protein level as well. Moreover, if possible, we wanted to evaluate the prognostic significance of ADAM9 expression in PDACs. In this study, we demonstrate that ADAM9 expression distinguishes PDACs from pancreatic acinar cell carcinomas and endocrine tumours. In addition, the cytoplasmic expression of ADAM9 has a prognostic potential.

## MATERIAL AND METHODS

### Patients and tissues

Formalin-fixed, paraffin-embedded tissue blocks were obtained from surgical specimens from 59 patients (mean age 59 years; range 31–76) with PDAC, who were operated at the Department of Visceral, Thoracic and Vascular Surgery, University Hospital Carl Gustav Carus, Technical University of Dresden, between 1996 and 2001. All PDAC patients received standard surgical therapy based on their clinical stages. In addition, tissue samples were obtained from surgical specimens from 32 patients with chronic pancreatitis, 11 patients with pancreatic endocrine tumours and 24 patients with acinar cell carcinomas. These tissues were selected from the institutional files and consultation files of the Department of Pathology, University of Kiel. Five patients with pancreatic endocrine tumours, one patient with acinar cell carcinoma and all 32 patients with chronic pancreatitis were operated at the Department of General Surgery and Thoracic Surgery, University of Kiel, between 1994 and 2002. All patients were randomly selected without stratification for known preoperative or pathological prognostic factors. The PDACs were staged (TNM classification) and reclassified histologically (JL, GK) according to the WHO classification (Kloppel *et al*, 2000). The clinicopathological features of the PDACs are listed in Table 1

**Table 1:** ADAM9 expression patterns in pancreatic ductal adenocarcinomas (PDAC) correlated with clinicopathological variables

		**ADAM9 expression**		**Luminal ADAM9**		**Basolateral ADAM9**		**Cytoplasmic ADAM9**	
	**Total no. of patients**	**+**	**−**	**+**	**−**	**+**	**−**	**+**	**−**
PDAC	59	58	1	57	2	32	27	17	42

*Histological grade*									
G1/2	33	32	1	32	1	9	18	4	29
G3	26	26	0	25	1	23	3	13	13

*Tumour stage*									
pT									
pT1	1	1	0	1	0	0	1	0	1
pT2	6	6	0	6	0	4	2	2	4
pT3	47	46	1	45	2	25	22	12	35
pT4	5	5	0	5	0	3	2	3	2
pN									
pN0	23	22	1	21	2	12	11	6	17
pN1	36	36	0	36	0	20	16	11	25
pM									
pM0	52	51	1	2	50	30	22	5	37
pM1	7	7	0	0	7	2	5	2	15

*Residual tumour*									
R0	42	41	1	2	40	27	15	13	29
R1	12	12	0	0	12	4	8	2	10
R2	5	5	0	0	5	1	4	2	3

*UICC stage*									
1	3	3	0	3	0	1	2	2	2
2	16	15	1	19	2	11	8	3	3
3	25	25	0	25	0	15	10	7	7
4a	6	6	0	6	0	4	2	4	4
4b	6	6	0	6	0	1	5	1	1

.

### Immunohistochemistry

Sections (4 *μ*m) were cut from formalin-fixed, paraffin-embedded pancreatic tissue. The sections were mounted on superfrost slides (Menzel Gläser, Braunschweig, Germany), dewaxed with xylene and were gradually hydrated. Antigen was exposed by heating the sections under high pressure in Tris–EDTA–citrate buffer for 3 min. The primary goat polyclonal anti-mouse ADAM9 antibody (AF949, R&D Systems, Wiesbaden, Germany), which crossreacts with human ADAM9, was diluted (15 *μ*g ml^−1^) in PBS containing 2% horse serum (Vector Laboratories, Burlingame, CA, USA). After incubation for 45 min, the reaction was detected with a biotinylated anti-goat antibody (5 *μ*g ml^−1^, Vector Laboratories) and avidin–biotin–peroxidase (ABC ELITE, Vector Laboratories). Diaminobenzidine served as chromogen. Afterwards, the slides were briefly counterstained with haematoxylin. For the negative control, the primary antibody was omitted. The staining intensity was evaluated semiquantitatively as negative, weak, moderate or strong. The final results, however, were recorded as positive (weak, moderate or strong staining) or negative staining only. In addition, the staining pattern was evaluated and a distinction was made between labelling of the luminal (apical) cell membrane of tumour cells forming glandular structures, the basolateral cell membrane and the cytoplasm. The staining was evaluated independently by two pathologists, who were unaware of patient survival.

### Statistical analysis

The Mantel–Haenszel test was used to assess the correlation between the clinicopathological findings and ADAM9 expression. For the survival analysis, a univariate analysis of 59 patients with PDAC was performed using the Kaplan–Meier method, Mantel–Haenszel estimation of the hazard ratios and log-rank tests for comparing between the strata (Peto *et al*, 1977). The histological grade, pTNM stage, R (residual tumour) stage and UICC stage were evaluated in a multivariate analysis using the Cox proportional regression hazard model. The differences at *P*<0.05 were considered significant. For statistical evaluation, the SAS/STAT software version 8 and SPSS software v. 11.0.1 were used.

## RESULTS

### ADAM9 immunostaining

**Figure 1:**
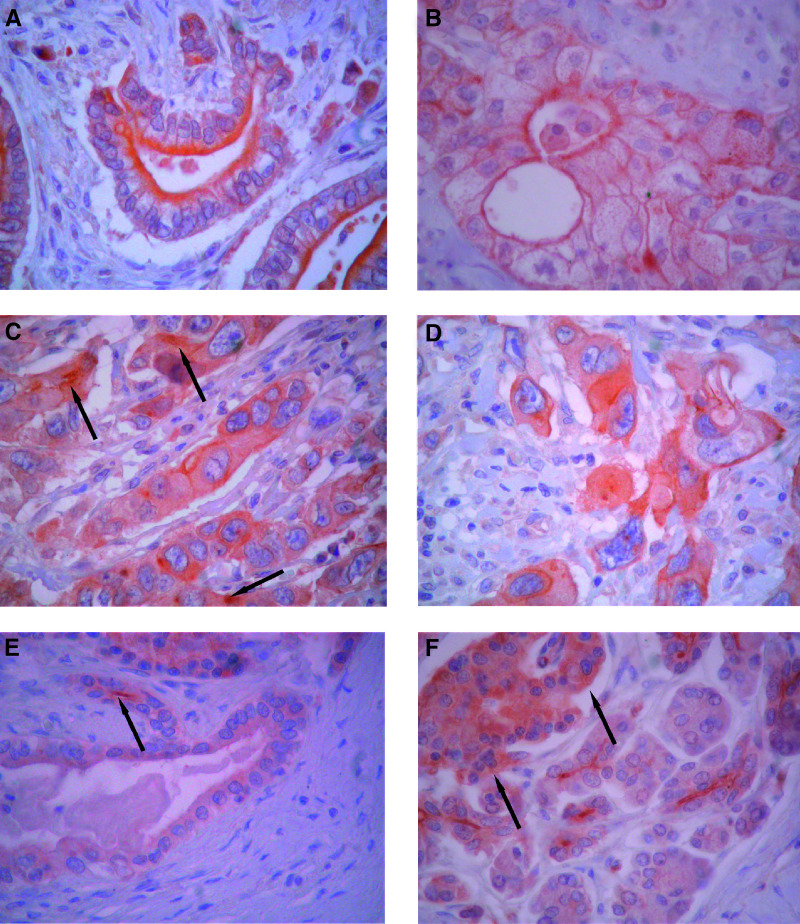
ADAM9 immunostaining in pancreatic tissues. Well-differentiated PDAC showing distinct apical ADAM9 staining at the luminal cell membrane (**A**); PDAC with luminal and basolateral membranous ADAM9 staining (**B**); poorly differentiated PDAC with basolateral membranous and focally accentuated cytoplasmic staining (arrow) (**C**, **D**); chronic pancreatitis with faint apical staining of the epithelium of a small duct (arrow) (**E**); normal pancreatic tissue showing staining of the luminal cell membrane of centroacinar cells and intralobular duct cells and faint granular staining of the cytoplasm of islet cells (arrow) (**F**).

Immunohistochemically, 58 of the 59 PDACs were positive for ADAM9 (Table 1, Figure 1A

). In 57 of the 59 PDACs, there was staining of the luminal cell membrane in areas with glandular formation. In 32 tumours, the membranous staining at the luminal side of the cell was accompanied by basolateral membrane staining. In 17 tumours, there was additionally strong cytoplasmic staining (Table 1, Figure 1B–D). Adjacent non-neoplastic pancreatic tissue as well as chronically inflamed pancreatic tissue showed weak ADAM9 expression along the luminal membrane of intralobular duct cells and centroacinar cells (Figure 1E and F). Hyperplastic and proliferative duct lesions (i.e. pancreatic intraepithelial neoplasiaFigure 1A and 1B) were negative. Occasionally, a few acinar cells showed weak cytoplasmic staining and the islet cells generally displayed weak granular cytoplasmic labelling (Figure 1F). All endocrine tumours of the pancreas lacked cytoplasmic expression of ADAM9. This was also true of acinar cell carcinomas, with the exception of two that showed weak membranous staining in areas with dilated acinar structures, so-called glandular formation.

### ADAM9 expression and patient survival

We found no significant association between cytoplasmic ADAM9 staining and patient age or stage, whereas the tumour grade was found to be statistically significant (*P*=0.03, Fisher's exact test; Table 2

**Table 2:** Relationship between cytoplasmic ADAM9 expression and various clinicopathological factors in all patients with pancreatic ductal adenocarcinomas (PDAC)

**Characteristic**	**All cases**	**Cytoplasmic ADAM9-negative**	**Cytoplasmic ADAM9-positive**	**Significance**
All PDACs	59 (100%)	42 (71%)	17 (29%)	

*Age at surgery*				0.15^a^
<61 years	28 (48%)	17 (61%)	11 (39%)	
>61 years	31 (52%)	25 (80%)	6 (20%)	

*Histological grade*				
G2	33 (56%)	29 (88%)	4 (12%)	0.03^a^
G3	26 (44%)	13 (50%)	13 (50%)	

*Tumour stage*				0.38^b^
pT1	1 (1,7%)	1 (100%)		
pT2	6 (10%)	4 (67%)	2 (33%)	
pT3	47 (80%)	35 (75%)	12 (25%)	
pT4	5 (8.3%)	2 (40%)	3 (60%)	
pN				0.78^a^
pN0	23 (39%)	17 (74%)	6 (26%)	
pN1	36 (61%)	25 (69%)	11 (31%)	
pM				1.00^a^
pM0	52 (88%)	37 (71%)	15 (29%)	
pM1	7 (12%)	5 (71%)	2 (29%)	

*Residual tumour*				0.75^a^
R0	42 (71%)	29 (70%)	13 (30%)	
R1	17 (29%)	13 (76%)	4 (24%)	
UICC stage				0.3^b^
I	3 (5%)	1 (33%)	2 (67%)	
II	16 (27%)	13 (81%)	3 (19%)	
III	25 (42%)	18 (72%)	7 (28%)	
IV	12 (20%)	7 (58%)	5 (42%)	

a Fisher's exact test.

b χ^2^ test.

). Similarly, no correlations between the intensity of ADAM9 staining, luminal ADAM9 and basolateral ADAM9 expression and these clinicopathological parameters were obtained. We also found no correlation between ADAM9 staining intensity and the occurrence of cytoplasmic ADAM9 staining (data not shown). For the univariate survival analyses, cumulative survival curves were calculated according to the Kaplan–Meier method. This analysis demonstrated statistical significance for the following parameters: tumour grade and cytoplasmic and basolateral ADAM9 expression (Figure 2

**Figure 2:**
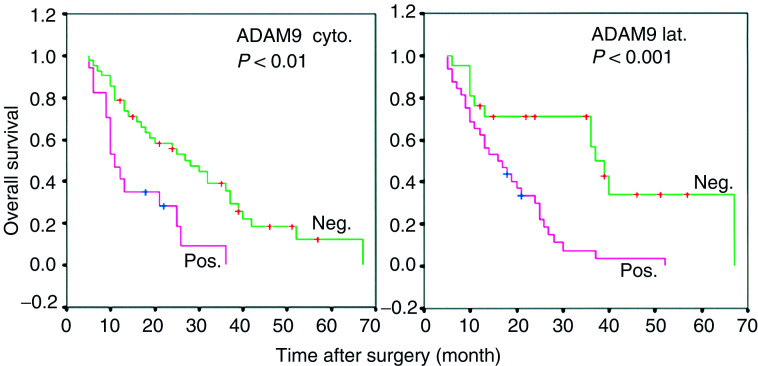
Kaplan–Meier curves of overall survival of PDAC patients showing different ADAM9 expression patterns (cyt: cytoplasmic; lat: basolateral).

, Table 3

**Table 3:** Results of the univariate and multivariate analyses in patients with pancreatic ductal adenocarcinomas (PDAC)

		**HR (95% CI)**		***P*-value**	
**Category**	**No. dead/total (%)**	**Univariate^a^**	**Multivariate^b^**	**Univariate**	**Multivariate**
Cytoplasmic ADAM9 expression					
Negative	32/42 (76.2)	1	1	–	–
Positive	15/17 (88.2)	3.94 (1.72 … 9.05)	2.85 (1.21 … 6.71)	<0.001	<0.05

Laterobasal ADAM9 expression					
Negative	12/21 (57.1)	1	–	–	–
Positive	30/32 (93.7)	3.49 (1.81 … 6.73)	–	<0.001	–

Grade					
1/2	21/33 (63.6)	1	1	–	–
3	26/26 (100.0)	4.81 (2.43 … 9.52)	3.34 (1.49 … 7.47)	<0.001	<0.01

a A total of 59 patients with PDAC.

b A total of 42 patients with curatively (R0) resected PDAC.

HR=hazard ratio.

). The mean survival time of patients with PDAC without cytoplasmic ADAM9 expression was 30 months (±3; median 28±4), compared to 16 months (±2; median 11±1) for those whose tumours showed cytoplasmic ADAM9 expression (*P*<0.001, Table 3). The mean survival time for patients with PDAC who showed no basolateral ADAM9 staining was 40 months (±6; median 37±3), compared to 18 months (±4; median ±2) for those PDAC patients with basolateral ADAM9 expression (*P*<0.001, Table 3).

A multivariate progression analysis based on the Cox proportional hazard model was performed in order to test the independent value of each parameter predicting overall survival in patients with R0 resection (*N*=42). Only cytoplasmic expression of ADAM9 and tumour grade were found to be independent prognostic factors for poor overall survival (cytoplasmic ADAM9: HR=2.85; 95% CI: 1.21–6.71, *P*<0.05; tumour grade: HR=4.81; 95% CI: 2.43–9.52; *P*<0.01) (Table 3).

## DISCUSSION

ADAM9, a member of the ADAM family that is involved in various biological processes (Moss *et al*, 2001), was found to be overexpressed in prostate carcinoma cell lines, hepatocellular carcinoma and breast carcinoma (McCulloch *et al*, 2000; Le Pabic *et al*, 2003; O'Shea *et al*, 2003). In PDAC, we and others observed ADAM9 overexpression by gene expression profiling. While Iacobuzio-Donahue *et al* (2003) used cDNA microarray analysis, with subsequent validation of the ADAM9 overexpression by RT–PCR in PDAC cell lines, we based our examination on the use of Affymetrix GeneChips and validated the distinct immunohistochemical expression of ADAM9 in a small series of PDACs (Grutzmann *et al*, 2003).

This study was designed to further validate the significance of ADAM9 overexpression in PDACs by comparing the results with those obtained in pancreatic tumours other than PDACs, and by correlating the immunohistochemical labelling of the individual PDACs with the survival of the patients.

ADAM9 was detected in 58 of 59 PDACs along the apical lumen-oriented membrane of the neoplastic glandular structures. In addition, 32 of the 59 PDACs showed staining of the basolateral cell membrane and 17 revealed cytoplasmic positivity. In the normal adjacent pancreatic tissue, ADAM9 staining, although weak, was also detected at the luminal surface of the interlobular ductal cells and centroacinar cells. It appears that ADAM9 is preferentially a luminal membrane-bound protein of duct-type pancreatic cells. As the apical membrane labelling, observed in the adjacent normal pancreatic tissue, is preserved in almost all PDACs, we might speculate that ADAM9 function could at least partly be maintained in the tumour cells. Among the pancreatic tumours, that is neoplasms of the acinar, endocrine and ductal phenotype, ADAM9 expression was selective for PDACs. Interestingly, endocrine neoplasms did not express ADAM9, although the islet cells in the normal pancreas displayed consistent granular cytoplasmic staining. These results indicate that ADAM9 might not play a role in the biology of nonduct-type neoplasms of the pancreas, but may be important for the biology of PDACs.

The distribution pattern of ADAM9 in PDACs was related to the differentiation of the individual tumours. More than two thirds of the well and moderately differentiated PDACs showed only apical membranous ADAM9 labelling, while poorly differentiated PDACs usually exhibited additional basolateral membranous and cytoplasmic staining. Whether this change bestows a progression advantage on the tumour cells is not yet known. However, because we found that cytoplasmic and basolateral ADAM9 staining correlates with poor survival in PDAC patients, it may be speculated that this over-expression pattern of ADAM9 promotes PDAC progression.

The relationship between the ADAM9 expression pattern in PDAC and the survival probability was tested in a series of curatively resected patients. This test, using a multivariate analysis, revealed cytoplasmic expression of ADAM9 to be an independent prognostic factor in patient survival. The second independent factor detected by this analysis was tumour grade, confirming earlier studies (Luttges *et al*, 2000). As there was a relationship between differentiation and the ADAM9 expression pattern, the possibility has to be considered that the two factors might be interrelated.

So far the precise function of ADAM9 in the pancreas is unknown. The designation ADAM is derived from their two transmembrane domains, which possess A Disintegrin And a Metalloprotease function (Izumi *et al*, 1998). The ADAMs are a multifunctional gene family, some members of which have been shown to play a role in diverse biological processes such as fertilization, myogenesis, neurogenesis and the activation of growth factors/immune regulators such as TNF-alpha. Moreover, ADAM9 is known to cleave heparin-binding EGF-like growth factor (Roghani *et al*, 1999). The disintegrin function probably relates to cell-to-cell and cell-to-extracellular matrix (ECM) adhesive interactions and transduction of signals from the ECM to the cell interior and *vice versa*. It may be involved in cell migration, invasion, intra- and extravasation and platelet interaction (Poggi *et al*, 1993; Mizejewski, 1999).

If ADAM9 overexpression is involved in PDAC progression, it may exert its action either via its disintegrin domain or its metalloproteinase domain or, most likely, via both. Various matrix metalloproteinases (MMP) like MMP2 and MMP9 have been described as being overexpressed in PDACs and seem to play an important role in the progression of PDAC (for a review cf. Bloomston *et al*, 2002). These observations led to a clinical trial of the metalloproteinase inhibitor marimastat in PDAC, which provided evidence of a dose response (Bramhall *et al*, 2001). Moreover, marimastat is potent not only against MMPs but also against ADAM9 (Moss *et al*, 2001). It may therefore be speculated that the response to marimastat in patients with PDAC may be in part due to inhibition of ADAM9. If this proved true, ADAM9 might play a role in tumour progression, and might be used not only for prognostic and diagnostic purposes but also for novel therapeutic approaches. Misallocation of ADAM9 from the luminal membrane to the cytoplasm and the basolateral membrane might add to an activation of growth factors and the degradation of ECM by ADAM9.

In conclusion, we have demonstrated that ADAM9 is overexpressed in PDACs but not in endocrine tumours or acinar cell carcinomas. Furthermore, we found a significant association between cytoplasmic ADAM9 overexpression and survival in patients curatively resected for PDAC. This suggests that cytoplasmic ADAM9 overexpression may be a useful diagnostic marker and could also become a potential target in the treatment of PDAC.
